# Awareness as inference in a higher-order state space

**DOI:** 10.1093/nc/niaa011

**Published:** 2020-05-10

**Authors:** Fleming Stephen M

**Affiliations:** Wellcome Centre for Human Neuroimaging, Institute of Neurology, 12 Queen Square, London WC1N 3AR, UK

*Neuroscience of Consciousness*, Volume 6, Issue 1, 2020, https://doi.org/10.1093/nc/niz020

The first version of this article mislabeled the references to Michel and Morales 2019 and Graziano 2013 on p. 2 and Brown et al. 2019 on p. 7. This is now corrected. The publisher regrets the error.

